# A unified signal of attentional control

**DOI:** 10.3389/fpsyg.2014.01056

**Published:** 2014-09-19

**Authors:** Olivier A. Coubard

**Affiliations:** ^1^The Neuropsychological Laboratory, CNS-FedParis, France; ^2^Laboratoire Psychologie de la Perception, UMR 8242 CNRS-Université Paris DescartesParis, France

**Keywords:** vision, attention, reward, primary visual cortex, animals

Recently, Stănişor et al. (2013) showed in monkeys that the reward value of a visual stimulus is a good predictor of primary visual cortex (area V1) activity. Furthermore V1 neurons with the strongest reward value effect were also the ones showing the strongest attention effect. Taken together, the authors concluded on overlapping reward value and top-down attention, and suggested that theories about reward and top-down attention coding on vision might be unified. Alternatively, we suggest that the unified signal for attention and reward is actually an attentional control signal originating in the prefrontal cortex (PFC).

Technically, it is not clear how the design by Stănişor et al. (2013) allows disentangling between attention and reward. Our observation is reminiscent with the critical review by Maunsell (2004) according to which neurophysiological studies aiming at dissociating attention and reward have failed to distinguish between the allocation of attention and the representation of the subject's assessment of what is to be rewarding. In this case, it is likely that neuronal signals related to the two cognitive processes may be found to be unified. Theoretically, it is not excluded that the two processes involved in Stănişor et al.' study (2013) may belong to a single cognitive function, namely attentional control. Attentional control is the ability to maintain goal-directedness by sustaining information-processing in the face of distraction. Associated with the activity of the PFC, it controls all subsidiary processes subserved by other cortical and subcortical regions. Primary sensory cortices, such as area V1, are also modulated by the PFC (Miller and Cohen, 2001).

Since seminal studies in humans by Harlow (1848/1999) and Luria (1966) on affective and cognitive processes of PFC, motivation and attention have traditionally been considered as distinct aspects of control. In line with Spinoza, Damasio (1994) unified the two processes showing that affect and its associated somatic markers are critical to make proper selection and decision. More recently, attentional control models have provided comprehensive descriptions of motivational and cognitive processes of control. As example, Stuss et al.'s model (Stuss et al., 1995) describes several attentional tasks (setting, sustaining, suppressing, switching, concentrating, preparing and sharing) recruiting multiple cognitive processes (energizing, inhibiting, adjusting, monitoring, controlling and task setting). Each of these processes is expected to fit a particular neural network, which to date has been identified for at least three of them. Energizing schemata or energization, the process of initiating and sustaining any response, is related to dorsomedial PFC, while monitoring the level of activity in schemata is related to right dorsolateral PFC (Stuss, 2011). Energization, involved in all attentional tasks except suppressing, may be viewed as motivational aspects of attention. Such modulation by the dorsomedial and dorsolateral PFC for respectively energization and monitoring act in parallel onto all brain areas, including sensory cortices.

In the meanwhile, theories of decision signal have described a random walk process determined by the distance between initial and final thresholds, which can be modulated by prediction or urgency, and the accumulation of sensory evidence or gain, which varies with the supply of information provided to the system (Carpenter, 1981; Hanes and Schall, 1996; Kim and Shadlen, 1999). Bridging attention and decision models, we additionally suggest that energization is not different from adjusting the distance to the decision signal thresholds, whereas monitoring may fit the accumulation of evidence. Consistent with this idea, Domenech and Dreher (2010) demonstrated double dissociation in humans in which the anterior cingulate cortex and dorsolateral PFC code, respectively, the distance to the threshold and the accumulation of sensory evidence. In this context, we suggest that monitoring and energizing may be the two signals recorded by Stănişor et al. (2013) for respectively attention and reward, consistent with their reported behavioral accuracy, eye speed, reaction time variability, and patterns of V1 neural activity. Thus two apparently distinct signals recorded in V1 that are dependent of the relative and of the attention value of the stimulus may be in fact the result of two attentional control processes. In other words, the reported unified signal for attention and reward can also be viewed as a double signal of a signal function—attentional control—in which energization and monitoring act as two modulatory systems of the attention network.

How could future studies be improved to separately examine attention and reward and their related neuronal activities? Our suggestion is twofold. About the task, one possibility to assess attention without reward might be to observe the subject' spontaneous movement to stimuli varying in different aspects (space, time, features, etc.). For reward, we would suggest to observe the subject's self-reward (using non-addictive substance) in relationship to spontaneous preferred movement to stimuli equal in all aspects thus not requiring attention. In summary, what may be observed is not subject's conditioning to human experimenter's expectations in terms of the importance given to one aspect of the stimulus (for attention) and of performance merit and credit (for reward), but rather the subject's spontaneous behavior and expectations at either attentional or motivational levels. About the measurement, one possibility to distinguish between attention and reward might be to use our knowledge of technical tools and of neural bases of Bayesian decision models. Indeed, it is likely that some modulation of the distance to thresholds versus of the gain might reveal some action onto motivational versus monitoring dimensions of attention, respectively.

## Conflict of interest statement

The author declares that the research was conducted in the absence of any commercial or financial relationships that could be construed as a potential conflict of interest.
